# Surgical Management of Recurrent Gastrojejunocolic Fistula With Gastric Antrectomy and Bilateral Vagotomy

**DOI:** 10.1155/cris/5078444

**Published:** 2026-05-21

**Authors:** Giancarlo Sticca, Michel Morin, Madeleine Poirier, Lucas Sideris, Yves Bendavid

**Affiliations:** ^1^ Department of Surgery, Division of General Surgery, University of Montreal, Montreal, Canada, umontreal.ca

## Abstract

**Background:**

Gastrojejunocolic fistulas (GJCFs) are rare complications following gastrojejunostomy and Roux‐en‐Y procedures, most often in the context of peptic ulcer disease.

**Case Report:**

A 42‐year‐old woman, with a history of cocaine addiction, underwent an gastrojejunostomy, a transgastric closure of the gastric pylorus, and a Graham patch for a perforated duodenal ulcer. She then presented with a dehisced gastrojejunal anastomosis. Resection of the dehisced anastomosis and Roux‐en‐Y reconstruction was performed. Eight months later, the patient returned to the hospital with a GJCF. A one‐stage en‐bloc GJCF resection with redo Roux‐en‐Y was performed. Six months later, a scan demonstrated a recurrent GJCF. Antrectomy and bilateral truncal vagotomy were performed to avoid recurrent gastric acid secretion. Resection of the dehisced gastrojejunal anastomosis and the segment of fistulized transverse colon was performed. A third Roux‐en‐Y reconstruction was fashioned. The patient evolved well and showed no signs of recurrent disease.

**Discussion:**

The cornerstone treatment of GJCF is the administration of parenteral nutrition, followed by a single‐stage en‐bloc procedure. Some factors that can contribute to recurrent GJCF are retained gastric antrum syndrome, incomplete vagotomy, and noncompliance with oral antacid therapy. Despite the significant leaps in the medical management of peptic ulcer disease in recent years, surgical strategies that reduce excess gastric acid secretion should be considered in select cases.

**Conclusion:**

This case highlights that gastric antrectomy and bilateral vagotomy have not been rendered obsolete in the 21st century, particularly in patients who cannot be compliant with oral proton pump inhibitors.

## 1. Introduction

Gastrojejunocolic fistula (GJCF) is a rare entity. The majority of GJCF cases are late chronic complications after gastrectomy and gastrojejunostomy for peptic ulcer disease [1–5]. Contributing factors include inadequate gastric resection or incomplete vagotomy [1–4, 6]. GJCF has become scarcer since the advent of proton pump inhibitors and eradication treatments for *H. pylori* [2]. The clinical triad is diarrhea, weight loss, and fecal vomiting [7, 8]. Nowadays, after ensuring adequate nutritional status, the cornerstone treatment is one‐stage en‐bloc GJCF resection with primary anastomosis [2, 3, 9]. We describe the case of a 42‐year‐old woman presenting to an academic center with recurrent complex GJCF. Written informed consent was obtained from the patient for publication of the case report. Risk factors, clinical presentation, diagnostic modalities, and management strategies are discussed.

## 2. Case Report

We present the case of a 42‐year‐old woman. She had an addiction to crack cocaine and a significant smoking history. Her surgical history was notable for an antecolic gastrojejunostomy, a transgastric closure of the gastric pylorus, and a Graham patch for a perforated duodenal ulcer performed at another institution. She had no other medical history and did not take any prescribed medications.

One month later, the patient was found in cardiac arrest in her residence. She was transferred to our institution. Resuscitative measures were successful. A CT scan demonstrated that the gastrojejunostomy had leaked. Urgent laparotomy was performed. The gastrojejunal anastomosis was resected, but the patient was hemodynamically unstable. Thus, the procedure was quickly ceased to allow for resuscitation at the intensive care unit, and the stomach and jejunum were left in discontinuity. The next day, the patient was brought back to the operating room, and the surgeon created a Roux‐en‐Y retrocolic gastrojejunal anastomosis. The anastomosis was performed on the posterior aspect of the stomach with healthy gastric tissue and avoided the mechanical staple line of the resected gastrojejunostomy from the day before. The Roux‐en‐Y limb was 25 cm long. At pathology, there was no evidence of antrum, ulceration, or neoplasia in the specimen. Through vasoactive support and long‐course antibiotic therapy, the patient recovered from her septic shock. She was treated with oral anticoagulant therapy for a pulmonary embolism. After 5 weeks of rehabilitation, the patient was discharged from hospital.

Eight months later, the patient presented with intractable feculent vomiting, diarrhea, and significant weight loss over the last 2 weeks. Volume resuscitation, vasopressors, and empirical antibiotic therapy stabilized the patient. Serum albumin was 15 g/L. A CT scan revealed a fistula between the stomach and the distal third of the transverse colon. Surgery was recommended after nutritional status optimization, which was then administered for 2 weeks. Gastroscopy demonstrated an intact gastrojejunal anastomosis without any evidence of fistulization or marginal ulcer. Rather, the fistula was observed to be 2 cm distal to the gastrojejunostomy, within the mechanical staple line of the resected gastrojejunostomy that had been left untouched during the previous Roux‐en‐Y. No ulcers or bleeding were visualized. Biopsy was negative for *H. pylori* and neoplasia.

The patient was consented for surgery. Laparotomy confirmed that the fistula stemmed from the partial gastrectomy staple line and fistulized with the transverse colon and jejunal Roux limb. This trifurcation confirmed GJCF. A one‐stage en‐bloc fistulectomy, including the gastrojejunal anastomosis, the Roux‐en‐Y limb, and 15 cm of transverse colon, was performed. A 20‐cm‐long Roux‐en‐Y was recreated with a hand‐sewn gastrojejunostomy and jejunojejunostomy. A hand‐sewn colocolic anastomosis was performed. On pathology, there was no evidence of antrum, ulceration, or neoplasia in the specimen. Postoperatively, a thoracic angioscan revealed a pulmonary embolism, which was treated with oral anticoagulant therapy. Three days postoperatively, significant free fluid and intestinal parietal thickening were observed on a scan. Given the patient’s hemodynamic instability, she underwent exploratory laparotomy; infected ascites was drained, but there was no evidence of anastomotic leak. The patient was discharged 10 days postoperatively.

Six months later, the patient was found unconscious at home after having episodes of abdominal pain, fecal incontinence, and diarrhea. The scan demonstrated a recurrently dehisced gastrojejunal anastomosis fistulizing with a voluminous retrogastric mass and with the splenic angle of the colon. Parenteral nutrition and broad‐spectrum antibiotics were administered for 10 weeks, after which she was scheduled for surgery. Laparotomy was performed. Antrectomy with truncal bilateral vagotomy was performed, including resection of the gastrojejunal anastomosis and the past Roux‐en‐Y limb, which was only 10 cm long. A third reconstruction with a Roux‐en‐Y limb measuring 50 cm was performed. A notable challenge was the necessity to create a terminolateral duodenojejunostomy at the base of the Roux‐en‐Y limb. The distal transverse colon was resected. An end colostomy with closure of the distal stump was performed because the transverse colon was ischemic due to the chronic inflammatory process. Additionally, the patient did not take the colic preparation preoperatively despite stating that she had been compliant. One week postoperatively, an abdominopelvic scan demonstrated nondrainable free fluid that was possibly infected. Empiric antibiotics were continued. A small subcutaneous collection was drained at bedside. A subsequent scan showed that the collection had significantly diminished. The patient was switched to oral antibiotics, tolerated oral diet, and was discharged from the hospital 4 weeks postoperatively.

The patient was seen in clinic 3 months postoperatively. Pathology confirmed the excision of the gastric antrum and the bilateral vagus nerves. The patient was asymptomatic and showed no signs of disease recurrence. A follow‐up scan confirmed no disease recurrence or postoperative complications.

## 3. Discussion

GJCF is a morbid pathology that often presents with diarrhea, weight loss, and fecal vomiting [7, 8]. Hypoalbuminemia, electrolytic abnormalities, and vitamin deficiencies are often present [5], all of which were identified in our patient’s lab work. A CT scan with oral and intravenous contrast demonstrates a diagnostic sensitivity of ~85%–90%, while endoscopy confirms fistulization in 70%–80% of cases; however, intraoperative exploration remains essential in complex or recurrent presentations [3, 4, 7, 8, 10]. Specifically, a CT scan may reveal direct visualization of the fistulous tract, enteric contrast in the wrong place, or abnormal adherence between the stomach, jejunum, and colon with loss of a fat plane. Endoscopy may reveal fistulous openings and defects around the anastomosis or staple line. Intraoperative exploration often confirms diagnosis through localization of a point of trifurcation between the stomach, jejunum, and colon, a defect at the staple line, or an inflammatory phlegmon. As such, preoperative imaging and endoscopic findings can be definitely correlated with intraoperative diagnostic criteria. In this case, intraoperative exploration confirmed the presence of GJCF. Similarly, Puia et al. [7] published a case series in which intraoperative exploration was necessary to diagnose GJCF in 16.7% of patients. Historically, two‐ or three‐stage procedures were necessary to address these types of fistulas [2, 3]: colostomy, fistulectomy, then colostomy reversal were often performed [2, 5]. In recent years, the administration of a minimum 4‐week course of parenteral nutrition, followed by a single‐stage en‐bloc procedure including GJCF resection and primary anastomosis, has been found to minimize morbidity and mortality [2, 3, 5, 9]. Similarly, the patient in this report underwent preoperative optimization with parenteral nutrition followed by a single‐stage procedure. In contemporary series, single‐stage en‐bloc resection following nutritional optimization is associated with reported recurrence rates below 10% and operative mortality rates under 5% [2, 3, 5, 9]. Severe hypoalbuminemia (serum albumin < 20 g/L) is commonly used as a threshold to mandate preoperative parenteral nutritional support prior to definitive surgical management [5, 7]. Even when performed after adequate preoperative nutritional optimization, en‐bloc resection of GJCF is associated with reported postoperative morbidity rates of ~20%, most commonly related to infectious, thromboembolic, pulmonary, and anastomotic complications [2, 3, 5, 9]. Postoperative monitoring in high‐risk patients should include close clinical follow‐up, early cross‐sectional CT scan, close nutritional follow‐up, and follow‐up endoscopy to promptly identify recurrence or septic complications [2, 3, 5, 9].

The etiology of the GJCF formation in this patient is important to consider. GJCF is mostly a late chronic complication after gastrectomy and gastrojejunostomy for peptic ulcer disease [1–5]. Despite marginal gastrojejunal ulceration often being the culprit for GJCF [1–4, 6, 10–12], our patient’s fistula stemmed 2 cm distally from the gastrojejunostomy and was found to be at the level of the partial gastrectomy staple line. Thus, the most likely etiology for fistula formation was a combination of ulceration of the gastric mucosa and a chronic partial gastrectomy staple line leak. To our knowledge, this is the first case describing such an etiology of GJCF in the literature.

Recurrent peptic ulcer disease after gastrojejunostomy is a challenge [13]. We hypothesized that four factors may have contributed to recurrent peptic ulcer disease and subsequent recurrent GJCF in this patient. First, gastrojejunostomies have a high risk of ulceration [13, 14] because the jejunal mucosa lacks the protective antacid mechanism, particularly if complete antrectomy and vagotomy are not performed. Second, performing truncal vagotomy has been shown to lower the chances of recurrent ulcers [1–4, 6, 8, 13] because it reduces stomach acid secretion. However, since the advent of proton pump inhibitors in the 21st century, it is more rarely practiced routinely. This case emphasizes that vagotomy may not have been rendered obsolete, despite the advances in pharmacotherapy in recent years. Third, retained gastric antrum syndrome may have contributed to this patient’s recurrent ulceration [1–4, 6, 13]. An anatomically retained antrum leads to increased gastrin levels and increased gastric acid secretion. Subsequently, ulceration recurs, most commonly at the gastrojejunal anastomosis. Surgically removing the antrum has been shown to significantly reduce the risk of recurrent peptic ulceration [8, 13]. During her first operation, antrectomy was not performed in this patient, likely due to the emergent nature of the intervention as well as the “damage control” strategy utilized. Fourth, GJCF and ulcers have become scarcer since the advent of proton pump inhibitors and eradication treatments for *H. pylori* [2]. However, due to the patient’s numerous addiction disorders, she could not be compliant with oral proton pump inhibitors when discharged from hospital. The patient’s significant smoking history was also a risk factor [1–4, 6]. In conclusion, meticulous surgical planning is required in recurrent GJCF, including identification of atypical fistula origins, exclusion of retained gastric antrum, and consideration of definitive acid‐reducing procedures to minimize recurrence. This case highlights that gastric antrectomy and bilateral vagotomy have not been rendered obsolete in the 21st century, particularly in patients who cannot be compliant with oral proton pump inhibitors.

## 4. Conclusion

GJCF often presents as a clinical triad of diarrhea, weight loss, and fecal vomiting. Preoperative optimization with total parenteral nutrition followed by single‐stage en‐bloc resection and primary anastomosis remains the cornerstone treatment. Factors that may contribute to recurrent GJCF are retained gastric antrum syndrome, incomplete vagotomy, and noncompliance with oral antacid therapy. Despite the significant leaps in the medical management of peptic ulcer disease in recent years, surgical strategies that reduce excess gastric acid secretion should be considered in select cases.

## Funding

No funding was received for this manuscript.

## Consent

Written informed consent was obtained from the patient for publication of clinical details, images, and discussion in accordance with institutional and journal ethical standards. A copy of the written consent is available for review by the Editor‐in‐Chief of this journal on request.

## Conflicts of Interest

The authors declare no conflicts of interest.

## Data Availability

The data that support the findings of this study are available from the corresponding author upon reasonable request.
